# Initiatives to boost resilience towards El Niño in Zimbabwe’s rural communities

**DOI:** 10.4102/jamba.v14i1.1194

**Published:** 2022-02-16

**Authors:** Jephias Matunhu, Stephen Mago, Viola Matunhu

**Affiliations:** 1 Department of Development Studies, Faculty of Commerce and Law, University of Fort Hare, Alice, South Africa; 2 Department of Development Studies, Faculty of Business and Economic Sciences, Nelson Mandela University, Gqeberha, South Africa; 3 Department of Public Administration, Faculty of Commerce and Law, University of Fort Hare, Bisho, South Africa

**Keywords:** El Niño, rural communities, livelihoods, resilience, poverty, Zimbabwe

## Abstract

Most Zimbabweans living in rural areas experience acute shortages of water for domestic and agricultural purposes. Household poverty amongst rural inhabitants is also increasing because of factors such as El Niño-induced droughts, overdependence on donor assistance and government’s failure to invest in sufficient water infrastructure. The purpose of this article is to interrogate the initiatives that have been taken to alleviate food insecurity in Zimbabwe’s rural communities. Under the spotlight are the strategies that rural communities and other stakeholders embraced to adapt to the effects of El Niño and to reduce food poverty. We used extensive literature review methodology and explorative qualitative design to investigate how rural communities and other stakeholders in Zimbabwe deal with the issues of food security in the context of persistent El Niño-induced droughts. The results show that rural communities in Zimbabwe continue to experience food security challenges that require collaboration between communities, government, non-governmental organisations (NGOs) and other stakeholders to build resilience against El Niño-induced droughts. Modernising water supply systems and agricultural management systems can improve the efficiency and effectiveness in food production and distribution.

## Introduction

Maxfield (2020) reported that globally, 800 million individuals are hungry and four million face critical water insecurity. The United Nations Sustainable Development Goal (UN-SDG) number 2 explicitly emphasises ending hunger and achieving food security. It also seeks to improve nutrition and promote sustainable agriculture (UN 2015). The SDG 6 calls for ‘increasing access to clean drinking water and sanitation for all’ (UN 2015). Previously, during the UN Conference on Sustainable Development (Rio+20) in 2012, world leaders reaffirmed that everyone has a right to safe and clean water and nutritious food. The UN Secretary-General’s call for the Zero Hunger Challenge was launched at Rio+20. The conference called on stakeholders such as governments, faith communities, civil society groups, research organisations (institutions), and the private sector to collaborate in order to end hunger and eliminate malnutrition. Article 15 of Zimbabwe’s Constitution requires the state to ensure production and storage of adequate food. Furthermore, the state should establish adequate food reserves and promote adequate and proper nutrition using mass education and other appropriate means. Article 77 of the Constitution explicitly recognises the right to enough food and water.

Seemingly, development practitioners, policy makers, environmentalists and disaster management practitioners are increasingly showing interest in the effects of El Niño events on human life. The purpose of this article is to determine the possible initiatives or strategies and support programmes that can boost food security in Zimbabwe’s rural communities. The article is organised as follows: after the literature review on El Niño and related aspects in section 2, research methodology used is provided in section 3, which is followed by results and discussion in section 4, and finally the conclusion in section 5.

## Literature review

### Theoretical reflection

This article is underpinned by a theoretical reflection on stakeholder engagement, whose tenets are collaboration in resource mobilisation, programming, and resilience building. These tenets are the hallmarks of sustainable climate proofing of smallholder farmers. Stakeholders (Wehn et al. 2018; White et al. 2018) are people or organisations with a positive interest in activities that promote social good. In the current context, stakeholder engagement is a process of identifying and bringing together people or organisations who are willing to collaborate in enhancing food security in the context of El Niño. Stakeholder engagement activities aim at building capacity, resilience and trust within the communities of interest (Burnside-Lawry & Carvalho 2016; Granville, Mehta & Pike 2016; Wehn et al. 2018; White et al. 2018). Stakeholder engagement also enhances adaptation, implementation (Sherman & Ford 2014) and success in coping with disruptive events. It is a critical mechanism for building strong relationships within communities (Granville et al. 2016).

Stakeholder engagement promotes the transfer of knowledge on El Niño and the food value chain amongst the stakeholders. The knowledge could radiate from government officials, non-governmental organisations (NGOs), food security experts, amongst other important stakeholders (Wehn et al. 2018). Stakeholder engagement is critical in harnessing resources, ideas, methods and strategies that are important for the recovery from the disruptive El Niño phenomenon. Communities prone to food and water insecurity can work towards building the capacity to recover from or resist the negative events (Burnside-Lawry & Carvalho 2016).

### The El Niño phenomenon

In sub-Saharan Africa, food poverty is linked to several factors, chief amongst them being insufficiency of water for industry, agriculture and domestic use. Water insufficiency is caused by hot and dry conditions created by the El Niño phenomenon. Extreme weather conditions affect crop production, thus reducing food supplies (Food and Agricultural Organization [FAO] 2016; Hao et al. 2019; Regional Inter-Agency Standing Committee [RIASCO] 2017).

The FAO (2016) further stated that:

The El Niño phenomenon poses a global threat to the agricultural livelihoods of millions of people. In Southern Africa, the impacts of El Niño have been felt across all sectors – food security, nutrition, agriculture, water and sanitation, energy, health and education – which leads to the suffering of vulnerable populations and to economic contraction. (p. v)

In addition, factors such as mismanagement of economies, disasters, conflicts, and poor agriculture strategies are linked to food poverty in the region.

El Niño refers to a climatic interaction linked to sea surface warming. The warming phase of the phenomenon (called El Niño-Southern Oscillation [ENSO]) stimulates drought conditions which become prevalent in Zimbabwe (Brazier 2015:6). ENSO creates both dry and hot conditions that negatively affect food crops (Hao et al. 2019). Household poverty in the rural areas of Zimbabwe is currently at 76% (Dube 2016) because of various factors such as El Niño-induced droughts (RIASCO 2017).

In Zimbabwe, El Niño is responsible for environmental fragility, severe livestock deaths and crop failure. For example, the 2015–2016 El Niño-induced drought in Southern Africa was the worst in 35 years (Hove & Kambanje 2019; Mazvimavi, Murendo & Chivenge 2017; World Food Programme [WFP] 2016). The phenomenon had widespread, deep and diverse impacts on food and nutrition security with more that 40 million people facing food insecurity resulting from crop failure (RIASCO 2017; WFP 2016). The 2015–2016 El Niño-induced drought crippled rainfed crop production in the agricultural sector (Arslan 2018; FAO 2016; Mazvimavi et al. 2017; RIASCO 2017; WFP 2016). Rainfed food crop production is the critical source of livelihood in Southern Africa (Mavhura, Manatsa & Mushore 2015). Outside the Southern African region, Owusu et al. (2019) reported crop failure and poor harvest in Ghana because of the 2015–2016 El Niño-induced drought. They also reported that ‘The major impacts of the El Nino induced rainfall failure and yield reduction were household food insecurity, loss of income, indebtedness and deepening poverty’ (Owusu et al. 2019:618). In Zimbabwe, the impact was significant because 70% of the food production is dependent on peasant agriculture (FAO 2016) and more than 80% of the rural households have no access to water for irrigation purposes.

**FIGURE 1:**
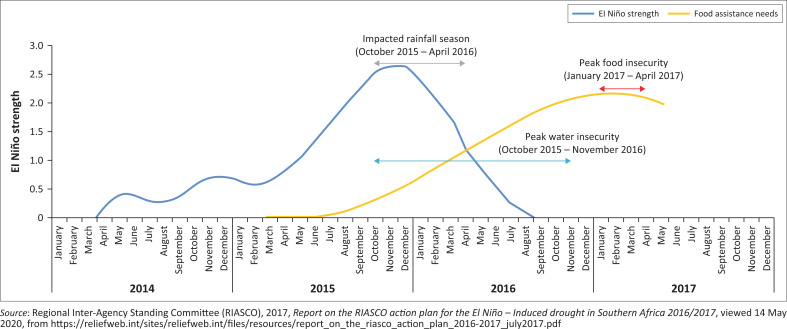
Southern Africa: Timeline of El Niño humanitarian impacts.

Figure 1 illustrates Southern Africa’s timelines of the El Niño phenomenon. The strength of El Niño, illustrated by a blue curve, shows how it impacted Southern Africa’s rainfall season (October 2015 to April 2016). This led to peak food insecurity between 2015 and 2017, thus causing food and water shortages leading to poverty and other downstream effects such as magnetron and increased number of school dropouts (RIASCO 2017; see also Hao et al. 2019).

RIASCO (2017) agreed that the 2015–2016 El Niño phenomenon created severe water and food shortages and worsened existing vulnerabilities. In addition, disease outbreaks were witnessed as people accessed drinking water from insecure sources (RIASCO 2017).

RIASCO (2017) further stated that hot and dry El Niño conditions create an environment ideal for the proliferation of Fall Armyworm (FAW), a pest which destroys crops. The FAW pandemic tends to hit harder on poorly resourced farming communities (Maluleke 2020). Furthermore, Maluleke (2020) lamented that ‘native African worms’ (NAW) are ravaging staple crops in the Southern Africa regions. Sound policies by governments, strategies, social safety nets, climate-smart agriculture programmes (RIASCO 2017), improved early warning systems (EWS) (Van Ginkel & Biradar 2021), better water management systems and natural resources management are factors to consider when dealing with food poverty.

Several policy related solutions to the challenges have been suggested for adoption by sub-Saharan governments. This article acknowledges the WFP’s (2016) proposal that investment in resilience building should be stepped up for sustainable rural agriculture in Southern Africa (Nhemachena et al. 2018). Resilience is the ability to withstand and recover from shocks and it is a vital element of adaptation (Mukwada & Manatsa 2017). The basket of resilience building would carry the following ingredients: upscaling social protection, agricultural reforms (to smart agriculture), climate change mitigation (Kumar 2014) (through smart livelihoods options), and management of natural resources. The major advantage of resilient building is that it empowers affected communities to recover, manage and survive future El Niño shocks with little or no external intervention.

**FIGURE 2:**
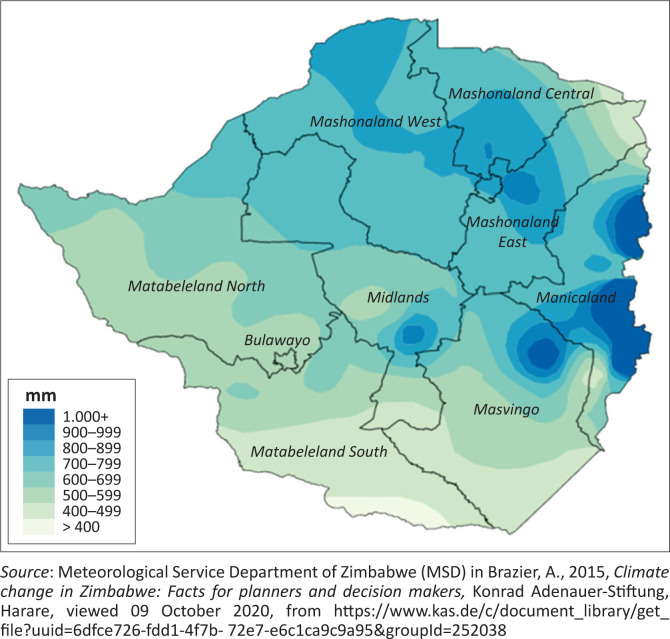
Zimbabwe average annual rainfall map.

Resilience refers to a community’s capacity to anticipate a shock or stress and respond to it with ease (Burnside-Lawry & Carvalho 2016; Dube 2020; Van Ginkel & Biradar 2021). Community resilience refers to the capability to renew, reorganise and restore situations after disruptive incidences (Burnside-Lawry & Carvalho 2016). Building resilience implies scaling up measures to reconstruct rural communities so that they are flexible, adaptable and open to learning because droughts permeate every aspect of human life (CARE International 2016; Catholic Relief Services (CRS) 2016; Van Ginkel & Biradar 2021). The study contributes towards building or strengthening absorptive, adaptive and transformative capacities of vulnerable rural communities in Zimbabwe where El Niño shocks are expected to intensify in the future. The study also contributes to the United Nations Sustainable Development Goals (UNSDG), Vision 2030 and Agenda 2063 which aim to mitigate the vulnerability of communities. In Zimbabwe, the El Niño phenomenon is closely associated with rainfall variability as shown in Figure 2.

The El Niño phenomenon worsens poverty in a country where 63% of the population lives below the ‘poverty datum line’ (PDL) and 27% of children below 5 years suffer stunted growth. As of November 2017, the PDL in Zimbabwe was $540.00 per family of six members (Mike Campbell Foundation 2019). Crop failure forces most households to become net food buyers amid grain price hikes of 60% in 2016 and 70% in 2017 (UN Zimbabwe 2016). Under these circumstances, most households in rural Zimbabwe resort to negative coping mechanisms such as limiting food portion sizes and/or reducing the meals taken per day.

Some families are not only insecure, but also suffer the lack of food sovereignty. In their Chipinge District (Zimbabwe) study, Chifamba et al. (2020) established that climate shock causes serious food deficits in the area, hence households choose to reduce the number of meals per day. Unfortunately, the strategies have not supported nutritional benefits amongst children.

In 2018, the Government of Zimbabwe needed to support about 302 000 households who were food insecure (Government of Zimbabwe 2017). This was even though the government had attained ‘the targeted maize production level of around 1.2 million tonnes in the 2016/17 Summer Cropping Season’ (Government of Zimbabwe 2017:156). Table 1 shows the disaggregated food insecurity across Zimbabwe’s 10 provinces. As illustrated in the table, food-insecure households in Zimbabwe’s 10 provinces have increased from 101 260 in July–September 2017 to 301 872 in January–March 2018. Masvingo province is top on the list with an increase of food-insecure households from 24 070 to 62 669, mainly because the area is known to be drought-prone.

**TABLE 1:** Food insecurity in Zimbabwe 2017.

Province	Number of households		
	July – September 2017	October – December 2017	January – March 2018
Manicaland	10 085	24 011	35 057
Mashonaland Central	8385	19 438	30 300
Mashonaland East	3170	10 415	22 744
Mashonaland West	24 463	37 886	48 020
Masvingo	24 070	46 150	62 669
Matabeleland North	7812	18 233	35 057
Matabeleland South	11 964	26 756	30 300
Midlands	11 311	23 841	22 744

**Total**	**101 260**	**206 730**	**301 872**

*Source*: Government of Zimbabwe, 2017, *National budget statement for 2018, ‘towards a new economic order’,* pp. 156–157, Ministry of Finance and Economic Development, Kampala.

In 2013, 76% of the rural households in Zimbabwe were considered poor and food-insecure (Mukwada & Manatsa 2017) because of Eli Niño-induced drought. The phenomenon also impacted negatively on rural households’ nutrition. In 2016, severe acute malnutrition (SAM) rate was 5.7%, the highest recorded in Zimbabwe since 2000 and surpassing the World Health Organisation’s (WHO) cut-off point of 5% (UN Zimbabwe 2016). ‘The SAM rate for children between 6 and 59 months was 2.1 percent, slightly above the 2 percent threshold for emergency response in Zimbabwe’ (UN Zimbabwe 2016:6).

El Niño is also a threat to the security of women who make up 52% of the Zimbabwe’s population which lives mainly (86%) in the rural areas (Echanove 2017). Over 70% of Zimbabwean women depend on rainfed agriculture, whilst 39% of rural households are women headed (UN Zimbabwe 2016). The El Niño disasters affect women’s livelihood options by negatively impacting on agricultural productivity. Droughts affect irrigation, thus affecting 83% of women engaged in irrigation schemes. They also reduce livelihood options for women who rely on casual paid labour on farms, irrigation schemes and agriculture-based industries.

Atmospheric warming (Tol 2005) affects agriculture production and increases livestock mortality from vector-borne diseases and heat stress. In the first quarter of 2016, 54% of the households in Zimbabwe had no regular water supply. Increasing water scarcity forced households to share unprotected water sources with livestock, thus increasing the risk of gastrointestinal infections.

## Research methods

Literature review is an appropriate method for carrying out an explorative qualitative study. Specifically, a traditional or narrative literature review process (Cronin, Ryan & Coughlan 2008; Green, Johnson & Adams 2006) was adopted for this study. Traditional or narrative literature review was used to provide a qualitative assessment of previously studied relationships amongst concepts or constructs (Blettner et al. 1999). Additionally, literature review has an advantage of limiting research bias because it constitutes a scientific narrative of the phenomenon being researched (Aliaga-Isla & Rialp 2013). For this present study, literature review enabled us to identify knowledge gaps in ways or initiatives for boosting resilience towards El Niño in Zimbabwe.

Although we did not use systematic literature review, we followed the following logical steps to improve the outcomes:

- Step1: Selection of the topic. We selected the topic ‘Initiatives to boost resilience towards El Niño in Zimbabwe’s rural communities’ with the methodology in mind.
- Step 2: Literature search strategy. In our search strategy, we used key words to develop search strings to improve the article extraction process. The search strings were guided by Boolean Operators to sharpen the search and exclude irrelevant articles in the search results. Search strings were developed from the key words and phrases such as El Niño, rural communities, adaptive capacity, resilience, poverty and Zimbabwe. The following search strings were used:
  1. (‘El Niño’ OR ‘droughts’ OR ‘dry conditions’ OR ‘hot conditions’ AND ‘food insecurity’ OR ‘food security’ AND ‘rural communities’ AND ‘Zimbabwe’)
  2. (‘El Niño’ OR ‘weather conditions’ OR ‘dry spells’ AND ‘crop failure’ AND ‘food security’ AND ‘Zimbabwe’)
  3. (‘El Niño’ OR ‘hot-dry conditions’ OR ‘Climate changes’ AND ‘food deficits’ OR ‘food shortages’ AND ‘resilience’ AND ‘livelihoods’ AND ‘Zimbabwe’)
  4. (‘community food resilience’ OR ‘resilience’ AND ‘indigenous knowledge systems’ AND ‘water harvesting’ AND ‘Zimbabwe’)
  5. (‘government agricultural support’ OR ‘government support’ OR ‘government intervention and food supply’ AND ‘food programmes’ AND ‘food security and children’ AND ‘Non-Governmental Organisations’)
- Step 3: Gathering relevant literature. This step involved reading abstracts to screen relevant articles. Some articles identified during the search process were included or excluded based on relevance. Further screening was done after reading full articles and thereafter irrelevant articles were excluded. Finally, the selected articles were analysed to provide answers to the pre-determined research objective.
- Step 4: Literature review write-up. After understanding the impact of El Niño and the initiatives used to cope with the circumstances, we then developed the literature section of the article.

We reviewed journal articles published in renowned and respectable journal databases such as Google Scholar, Taylor & Francis, Elsevier, Emerald Insight, Springer, AOSIS Publishing and African Journals Online (AJOL). Additional sources were drawn from grey literature which included policy documents, reports from FAO, WFP and RIASCO, the Ministry of Environment, Water and Climate (MEWC) and the Climate Change Management Department.

### Ethical considerations

This article followed all ethical standards for research without direct contact with human or animal subjects.

## Results and discussion

The purpose of this article was to explore the possible strategies and support programmes that rural communities in Zimbabwe can adopt to mitigate the effects of El Niño-induced droughts and boost food security. Food insecurity in rural communities is mainly caused by the El Niño phenomenon which induces food poverty. The initiatives identified in literature include: food resilience building in communities, indigenous knowledge systems, water harvesting systems, government agricultural support programmes, food security amongst children and interventions by non-governmental organisations.

### Food resilience building in communities

Zimbabwe is generally endowed with a resilient population (Brazier 2015). Social networks, especially in the rural areas help the rural population to cope with food supply shocks and stresses. Traditionally, the rural farmers of Zimbabwe used mixed crop–livestock systems which enabled them to take advantage of drought resistant animals such as goats and donkeys and small grain crops such as *rapoko* (finger millet), sorghum and *mhunga* (local Shona language term for pearl millet or bulrush millet). The general failure of these practices prompted the African Climate Summit of 16 November 2016 which identified seven (7) initiatives to enhance resilience in vulnerable rural communities (MEWC 2016). Chief amongst these initiatives is the Rural Resilience Initiative (RRI), which intended to build the capacities of rural communities to cope with climate change.

### Indigenous knowledge systems

The rural communities should make use of indigenous knowledge to enhance and ensure crop and livestock production. For example, rural communities use traditional medication such as *murunjurunju* or *muvengahonye* (*Cissus quadrangularis*) for livestock wound management (Marume et al. 2017). Unfortunately, these methods are not environmentally friendly as they involve debarking trees for medicine amid low aptitude towards re-afforestation. The other challenge is that the livestock treatments are not suitable for new animal and crop diseases. For this reason, the treatment regimens have a high failure rate. Scientific knowledge is required to help the farmers build a science-based innovative resilience mechanism against El Niño-induced droughts. Slow uptake of scientific knowledge weakens the communities’ capacity to cope with climate related food disasters.

### Water harvesting

In-field rainwater harvesting technologies are important to support underground water system and limit the impact of drought (Dile, Karlberg, Temesgen & Rockström 2013; Gwenzi & Nyamadzawo 2014). Wuta et al. (2017) stated that the rainwater harvesting mechanisms which are currently used include infiltration pits, ridges and basins which farmers dig along contours ridges. These in-field technologies for rainwater harvesting are currently being used in Zimbabwe (Wuta et al. 2017) at a low scale.

The Zimbabwean government has made efforts to put in place resilient water management systems to enhance food crop productivity. Zimbabwe’s MEWC introduced ‘Command Rain Water Harvesting Programme’ to boost agricultural water supplies in the rural areas. The Government has also put in place many irrigation schemes to enhance crop production in dry areas (Dube 2016; Moyo et al. 2017). Recently, the Government of Zimbabwe commissioned Tokwe Mukorsi Dam, the largest inland water body in Zimbabwe. With a carrying capacity of 1 915 000 m^2^, it is expected to benefit 3200 rural households. Dams such as Lower Gweru, Mushandike, Stanmore, Rupike, Chinyamatumwa, Mashoko, Rozva and Shereni support small scale irrigation schemes. Each of the schemes benefit an average of 180 households of six members each translating into 7680 rural beneficiaries. The list of irrigation schemes excludes small scale irrigation schemes such as Mabwematema in Zvishavane and others run by individuals using either underground water or small dams.

### Government agricultural support programmes

In 2016, the Government of Zimbabwe imported 700 000 metric tonnes of maize to reverse the impact of crop failure (The Herald 2016). Command agriculture was put in place to boost crop production in the rural farming sector. Command agriculture is a government and private sector-sponsored subsidy facility where farmers receive loans in the form of seeds, herbicides, pesticides, fertilisers, fuel and equipment. Under the programme, loan repayment is made using part of the harvest obtained in the following agriculture season. In 2016, Simpson Mukari from rural Goromonzi testified to the Integrated Regional Information Networks (IRIN) as follows:

I cultivated 20 hectares under command agriculture and what I harvested exceeded my expectations. I have repaid the loan in full, set aside enough maize to last a year for my [*six-member*] family, and might not need another loan since I made a good profit. After repaying the loan I was left with a net harvest of 40 tonnes of maize worth more than $15,000. (The New Humanitarian 2017:1)

In 2016, the scheme involved 2000 farmers and each farmer was expected to produce at least 1000 tonnes of maize. In 2017, the first batch of 200 heifers was handed over to 66 rural cattle farmers in Matabeleland (both North and South) provinces. This was under the $300 million livestock, wildlife and fisheries command programme. The Government provided $80 million and $220 million coming from the private sector. Under the poultry command programme, Irvines, a major poultry company in Zimbabwe, contributed 20 000 chicks including feed and vaccines to 15 farmers in Goromonzi District in Mashonaland East, 20 000 in Mazowe in Mashonaland Central, and 20 000 in Zvimba District in Mashonaland West (The Herald 2020).

### Food security amongst children

El-Nino-induced droughts have had serious repercussions on child nutrition in Zimbabwe. Over the years, the government has made concerted efforts to ameliorate this crippling condition. In fact, over a quarter of Zimbabwe’s children experience stunted growth because of food shortages and malnutrition (UNICEF Zimbabwe 2018). Stunting slows down growth and brain development amongst children. It also affects their performance at school and has long term effects in life. Studies have shown that one in every four children between the ages of 6 to 59 months suffer from vitamin A deficiency. In addition, 72% of the children live with iron deficiency, and 33% suffer from anaemia. Amongst women of child-bearing age, 25% have vitamin A deficiency, 60% have iron deficiency and 26% are anaemic. The Government of Zimbabwe has made efforts to address these challenges, chief amongst them was the introduction of the integrated management of acute malnutrition services in more than 97% of health care facilities and the National Food Fortification programme which saw the reduction of stunting from 33% in 2010 to 26% in 2018 (UNICEF Zimbabwe 2018).

### Non-governmental organisations interventions

Non-governmental organisations help in building resilience by funding community projects that alleviate water shortages. Water harvesting is an important strategy that reduces water supply shortages. According to Makwanya (2016:1) ‘rain-water harvesting subscribes quite well to the concept of Integrated Water Resource Management (IWRM)’. Christian Care, an NGO in Zimbabwe, helped Matebeleland North communities to construct tanks for water harvesting. Zimbabwe Project Trust (ZimPro), a local NGO, also helped Insiza District communities with rainwater harvest tanks to alleviate water shortages experienced in the area. Conservation Agriculture (CA) is one sustainable agricultural strategy implemented by NGOs such as Cooperative for Assistance and Relief Everywhere (CARE) to support food productivity and reduce poverty amongst rural communities (Kunzekweguta, Rich & Lyne 2017). The importance of CA is endorsed by Brazier (2015) who noted that livelihoods of rural communities in Zimbabwe are closely linked to the environment and climatic conditions.

Thierfelder et al. (2017) opined that CA is one of the ‘climate smart’ technologies for enhancing crop production in low rainfall regions. The FAO works with Zimbabwe’s Agricultural Extension Services (AGRITEX) and numerous NGOs to support the uptake of CA. Some of these NGOs include: CARE International and Hope Tariro (promoting CA in Masvingo), Lutheran Development Services, Oxfam, Practical Action and ZERO. In 2016, for example, CARE Zimbabwe supported more than 377 000 people in drought-hit areas. In addition, 17 271 pregnant and lactating women benefited from the emergency cash and food handouts provided by CARE Zimbabwe. The NGO is also running a 5-year project meant to help vulnerable people in the rural areas of Zimbabwe. About 271 000 vulnerable people are part of the project. The project covers the ‘areas of maternal health and nutrition, disaster risk management and resilience, climate smart agriculture and economic development’ (CARE 2016:1).

On the other hand, FAO’s 2016–2017 plans provided US$13 million for agricultural production, US$15.2 million to promote livestock production and US$6 million to promote access to water in vulnerable communities (FAO 2016). What is currently missing is effort by consortia of diasporas to support initiatives by the above-mentioned players. Table 2 shows the Zimbabwe United States Agency for International Development (USAID) Humanitarian Funding support which is under the Southern Africa Response support programme. The information demonstrates how NGOs are helping communities to cope with the circumstances created by El Niño-induced dry-hot events.

**TABLE 2:** USAID humanitarian funding for the Southern Africa response in financial year 2017.

Organisation	Activities	Districts	Budgeted funds
**CARE**	Agriculture and food security. Economic recovery and market systems, WASH	Bikita, Chivi, Zaka	$1 453 438
**CRS**	Agriculture and food security. Economic recovery and market systems	Bulima, Gwanda, Insiza, Umzingwane	$1 745 770
**FAO**	Agriculture and food security. Humanitarian contribution and information management	Bulima, Chivi, Gwanda, Insiza, Mberengwa, Mwenezi, Umzingwane and Zvishavane	$2 191 288
**IMC**	Nutrition, WASH	Beitbridge, Bubi, Insiza	$1 427 287
**IRC**	Agriculture and food security.	Chipinge, Chiredzi	$984 778
**Oxfam**	Agriculture and food security, WASH	Gutu, Matobo	$1 140 620
**SC/US**	Nutrition, WASH	Binga, Kariba, Mbire	$998 654
**UNICEF**	Nutrition, WASH	Binga, Hwange, Kariba, Nkayi, Umzingwane	$1 219 000
**World Vision**	Agriculture and food security, WASH	Binga, Kariba, Hwange, Mbire	$1 056 977

**Total USAID/OFDA funding for Zimbabwe $12 217 812**			

*Source:* USAID, 2017, *Southern Africa – Disaster response: Fact sheet #10, financial year (FY) 2017,* viewed 17 January 2022, from https://reliefweb.int/report/zimbabwe/southern-africa-disasterresponse-fact-sheet-10-fiscal-year-fy-2017.

USAID, United States Agency for International Development; OFDA, Office of U.S. Foreign Disaster Assistance; UNICEF, United Nations Children’s Fund; IMC, International Medical Corps; IRC, International Rescue Committee; FAO, Food and Agricultural Organisation; CRS, Catholic Relief Services; SC/US, Save the Children/U.S; WASH, water, sanitation and hygiene.

As illustrated in Table 2, NGOs such as CARE, CRS, FAO, United Nations Children’s Fund (UNICEF), International Rescue Committee (IRC), Oxford Committee for Famine Relief (OXFAM) and World Vision are working tirelessly to ensure water and food security in Zimbabwe. The activities supported by these organisations are important in fighting water and food insecurity, thus building resilience amongst communities in rural Zimbabwe. A total funding of $12 217 812 was budgeted to enhance resilience amongst rural communities in the face of El Niño conditions. These efforts are not enough to cushion all rural communities from El Niño-induced disasters; nevertheless, they are a step in the right direction.

## Conclusions

This research interrogated initiatives taken in Zimbabwe to create sustainable solutions for food security of rural household in the context of the El Niño phenomenon. Rural communities, the government and NGOs are seized with projects that aim to ameliorate the food security situation in rural communities impacted by the devastating effects of El Niño. Lack of proper coordination of efforts by the stakeholders affects the effectiveness of the food security initiatives. The study recommends the modernisation of the traditional agricultural management systems, investment in indigenous knowledge systems, intensive training of the rural communities on climate-proofing agriculture and commercialisation of rural agriculture. The government is encouraged to implement an appropriately mechanised and monitored command agriculture and command rainwater harvesting system. The success of the proposed systems will depend on the training of rural farmers and extension system, accurate weather information sharing and deep involvement of the diaspora, NGOs, the AGRITEX department in the MEWC, local communities, and research institutes in the country.
